# Thermal Imaging Reliability for Estimating Grain Yield and Carbon Isotope Discrimination in Wheat Genotypes: Importance of the Environmental Conditions

**DOI:** 10.3390/s19122676

**Published:** 2019-06-13

**Authors:** Sebastián Romero-Bravo, Ana María Méndez-Espinoza, Miguel Garriga, Félix Estrada, Alejandro Escobar, Luis González-Martinez, Carlos Poblete-Echeverría, Daniel Sepulveda, Ivan Matus, Dalma Castillo, Alejandro del Pozo, Gustavo A. Lobos

**Affiliations:** 1 Department of Agricultural Sciences, Universidad Católica del Maule, Curicó P.O. Box 684, Chile; 2 Plant Breeding and Phenomic Center, Faculty of Agricultural Sciences, Universidad de Talca, Talca P.O. Box 747, Chile; anmendez@utalca.cl (A.M.M.-E.); mgarriga@utalca.cl (M.G.); festrada@alumnos.utalca.cl (F.E.); escobar.opazo@gmail.com (A.E.); l.gonzalez.m@ieee.org (L.G.-M.); adelpozo@utalca.cl (A.d.P.); 3 Department of Viticulture and Oenology, Stellenbosch University, Matieland 7602, South Africa; CPE@sun.ac.za; 4 Centro de Investigación y Transferencia en Riego y Agroclimatología (CITRA), Talca P.O. Box 747, Chile; dsepulveda18@gmail.com; 5 Centro Regional Investigación Quilamapu, Instituto de Investigaciones Agropecuarias, Chillán P.O. Box 426, Chile; imatus@inia.cl (I.M.); dalma.castillo@inia.cl (D.C.)

**Keywords:** remote sensing, phenotype, phenotyping, phenomics, *Triticum aestivum*, water deficit, stress, infrared

## Abstract

Canopy temperature (Tc) by thermal imaging is a useful tool to study plant water status and estimate other crop traits. This work seeks to estimate grain yield (GY) and carbon discrimination (Δ^13^C) from stress degree day (SDD = Tc − air temperature, Ta), considering the effect of a number of environmental variables such as the averages of the maximum vapor pressure deficit (VPDmax) and the ambient temperature (Tmax), and the soil water content (SWC). For this, a set of 384 and a subset of 16 genotypes of spring bread wheat were evaluated in two Mediterranean-climate sites under water stress (WS) and full irrigation (FI) conditions, in 2011 and 2012, and 2014 and 2015, respectively. The relationship between the GY of the 384 wheat genotypes and SDD was negative and highly significant in 2011 (r^2^ = 0.52 to 0.68), but not significant in 2012 (r^2^ = 0.03 to 0.12). Under WS, the average GY, Δ^13^C, and SDD of wheat genotypes growing in ten environments were more associated with changes in VPDmax and Tmax than with the SWC. Therefore, the amount of water available to the plant is not enough information to assume that a particular genotype is experiencing a stress condition.

## 1. Introduction

Since the 1960s, crop temperature has been recognized as an indicator of water status [1]. When the plant is facing a water deficit, the stomata begin to close, reducing the transpiratory capacity (evaporative cooling) [2] and this results in increases in canopy temperature [3,4,5,6,7].

The development of infrared sensors/cameras has allowed a faster characterization of canopy temperatures [8]. At the same time, through computational analysis, it is possible to split the different parts of the image (e.g., soil, air, leaves, weeds) focusing only on the fraction(s) of interest [4,9,10]. Although thermal imaging does not directly measure stomatal conductance, the stomatal response is the main cause of changes in canopy temperature [10], so it is a useful tool to indirectly study spatial and temporal heterogeneity of leaf/canopy transpiration and the photosynthetic rate [10,11,12]. Indeed, in bread wheat grown in hot environments in Mexico under irrigation, a high correlation has been reported between temperature depression (TD = Ta − Tc) and leaf stomatal conductance (*r* = 0.76 − 0.85) and grain yield (GY; up to *r* = 0.84) [11,13]. Other researchers have used the concept of stress degree day (SDD), defined as the difference between leaf/canopy temperature (Tc) and air temperature (Ta) (SDD = Tc − Ta), which is equivalent to TD (but with positive values), mostly because canopy temperature in rainfed environments is lower than air temperature.

The main problem with the use of thermal assessments to estimate physiological and agronomic traits is that Tc is influenced by several environmental factors, such as air temperature and humidity, wind speed, net radiation, and soil water content [14,15,16,17]. Therefore, without detailed information about environmental factors, measurements of Tc are not sufficient to properly perform agronomic or physiological trait estimations.

Unlike irrigated conditions, a good correlation between Tc and GY under water deficit is not always expected [18]. However, it would be very useful for breeding programs to find such associations in stressful environments because the focus is on developing drought-tolerant cultivars with higher GY under water-limiting conditions.

It has been established that measurements of carbon isotope discrimination (Δ^13^C) in wheat are crucial for the selection of individuals with efficient water-use, mainly because this parameter is positively correlated with GY and negatively correlated with water-use efficiency (WUE) in moderately water-stressed to non-water-stressed environments [19,20,21,22,23,24,25]. The determination of Δ^13^C is simple and relatively fast but needs expensive equipment or engagement of a paid analysis service; attempts have also been made to estimate Δ^13^C by modeling the canopy spectral reflectance [24,26]. Under non-stressed conditions, the stomata remain open and the substomatal cavity is enriched with ^12^C relative to the air; the heavier isotopic ^13^CO_2_ has a lower diffusion speed than the lighter ^12^CO_2_ [20]. Additionally, the ribulose bisphosphate carboxylase/oxygenase (RUBISCO) carboxylation enzyme in C3 plants has a higher affinity to ^12^CO_2_. On the other hand, when stress forces the stomata to close, the proportion of ^12^CO_2_ in the substomatal cavity is reduced, thus increasing the amount of fixed ^13^CO_2_ [20]. Thus, daily conditions throughout the season will be summarized in the Δ^13^C of leaves and kernels (calculation details in Section 2.2.1). In this sense, under the expected climate change scenarios predicted for the coming decades [27], the estimation of Δ^13^C should be relevant in plant breeding programs oriented to environmental constraints [28,29,30].

Like all species, the phenotype of wheat plants is controlled by a large number of genes, and the expression of these is modulated, predominantly, in response to the environmental conditions (GxE) [31,32,33]. Consequently, it was hypothesized that the environmental conditions during and between seasons could interfere with the ability of canopy thermal imaging to estimate GY and Δ^13^C; in particular, the vapor pressure deficit (VPDmax) and soil water content (SWC), which can have a strong influence on canopy temperatures [34]. Therefore, the aim of this work was to study the reliability for estimating grain yield and carbon isotope discrimination in wheat genotypes growing under water stress (WS) and full irrigation (FI) conditions using thermal images, considering the relevance of the prevailing environmental conditions in estimation of the results.

## 2. Materials and Methods

### 2.1. Plant Material and Experimental Conditions

During four growing seasons (2011, 2012, 2014, and 2015), two sets of plant material were evaluated in two Mediterranean environments: (1) Cauquenes (c) (35°58’ S, 72°17’ W; 177 m.a.s.l.) under WS (rainfed) conditions during seasons 2011, 2012, and 2015, and under FI in 2015; and (2) Santa Rosa (sr) (36°32’ S, 71°55’ W; 217 m.a.s.l.) under WS in 2011 and 2015, and FI conditions in 2011, 2012, 2014, and 2015. Each combination of season (year), location (c or sr), and water condition (WS or FI) was considered as an environment.

A collection of 384 advanced lines and cultivars of spring bread wheat (*Triticum aestivum* L.), were evaluated during 2011 and 2012. Plant material originated from three breeding programs: the Instituto de Investigaciones Agropecuarias in Chile (INIA-Chile) (153 genotypes), INIA-Uruguay (178 genotypes), and the International Wheat and Maize Improvement Centre CIMMYT (53 genotypes). In 2014 and 2105, a subset of 16 genotypes with contrasting tolerance to water deficit was studied.

Each genotype was established in plots of five rows (2.0 × 0.2 m) with a seeding rate of 20 g m^−2^. Plots were fertilized with 260 kg ha^−1^ of ammonium phosphate (46% P_2_O_5_ and 18% N), 90 kg ha^−1^ of potassium chloride (60% K_2_O), 200 kg ha^−1^ of Sul-Po-Mag (22% K_2_O, 18% MgO, and 22% S), 10 kg ha^−1^ of Boronatrocalcita (11% B), and 3 kg ha^−1^ of zinc sulfate (35% Zn). Fertilizers were incorporated with a cultivator before sowing. During tillering, an extra 153 kg ha^−1^ of N was applied. Weeds were controlled with the application of flufenacet + flurtamone + diflufenican (96 g a.i.) as pre-emergence and a further application of MCPA (525 g a.i.) + metsulfuron-methyl (5 g a.i.) as post-emergent [35]; dates of sowing and the main phenological stages are shown in Table 1. Furrow irrigation was used at Santa Rosa, with the WS trials including one irrigation at the end of tillering (Zadocks stage 21–Z21; [36]) and FI comprising irrigations at the end of tillering (Z21), the flag leaf stage (Z37), heading (Z50), and early grain filling (Z71). At Cauquenes, the WS trials were purely rainfed and the FI trial during 2015 received sprinkler irrigation at Z37, Z50, and Z71. Approximately 50 mm was applied during each furrow/sprinkler irrigation application.

At each location, soil volumetric content (m^3^ m^−3^) was monitored periodically using 10HS sensors (Decagon Devices, Pullman, WA, USA), scanning the first 50 cm depth every 4 h. In order to generate the soil water content (SWC; mm), the volumetric values were multiplied by the soil depth (500 mm). Precipitation (mm), ambient temperature (°C), and relative humidity (%) were monitored hourly by autonomous weather stations (AWSs) belonging to the Red Agroclimática Nacional (National Agroclimatic Network, available at: www.agromet.inia.cl). Vapor pressure deficit (VPD; kPa) was determined hourly by the use of ambient temperature and relative humidity, according to Reference [37]. For analysis purposes, each environmental variable was studied as follows from sowing to harvest: (1) precipitation: daily summation; (2) ambient temperature: average of the daily maximum temperatures (Tmax); and (3) VPDmax: estimated at the highest ambient temperature and the corresponding relative humidity of each day, and then the average of the daily maximum VPDs (VPDmax) was calculated. Because water deficit in Mediterranean environments is present, primarily, between anthesis to grain filling, SWC was considered as the average of the daily mean values between anthesis and grain maturity.

Tmax, VPDmax, and SWC are summarized in Table 1 and Figure S1, and rainfall in Figure S2.

### 2.2. Evaluations

#### 2.2.1. Grain Yield and Carbon Isotope Discrimination

Grain yield was evaluated by harvesting the whole plot (2 m^2^) and was expressed as t ha^−1^. Carbon isotope composition (δ^13^C) was determined in mature kernels using an elemental analyzer (ANCA-SL, PDZ Europa, UK) coupled with an isotope ratio mass spectrometer, at the Laboratory of Applied Physical Chemistry at Ghent University (Belgium): δ^13^C (‰) = (^13^C/^12^C)_sample_/(^13^C/^12^C)_standard_ − 1 [20], where the ^13^C/^12^C ratio of the sample refers to plant material and the ^13^C/^12^C ratio of the standard is calibrated against the international standards from Iso-Analytical (Crewe, Cheshire, UK). The carbon isotope discrimination (Δ^13^C) of kernels was calculated as: Δ^13^C (‰) = (δ^13^C_a_ − δ^13^C_p_)/[1 + (δ^13^C_p_)/1000], where a and p refer to air and the plant, respectively [20]. δ^13^C_a_ from the air was taken as −8.0‰.

#### 2.2.2. Thermography

Thermal infrared images were taken using a portable infrared camera (i40, FLIR Systems, Sweden), at the soft dough (Z85) phenological stage. This camera provides images of 120 × 120 pixels (every pixel shows a temperature value) and has an uncooled infrared detector (microbolometer) in the spectral range from 7.5 to 13 microns. Infrared images were taken at ±2 h from the zenith (12:00 to 16:00 h), at a position of 45° from the horizontal, 0.5 m above the plant canopy, and a 3 m distance from the plot. Images were filtered using a process of interactive segmentation to exclude foreign matter from the picture (i.e., soil, weeds, neighboring plots, and air) using a custom MATLAB code [38]. To avoid surrounding plot noise, only the center of the image (30 × 30 pixels) was analyzed with a temperature frequency histogram (percentile level). The hottest and coldest pixels were eliminated, taking as a threshold the percentiles 1 downwards and 97.5 upwards, respectively. The remaining pixels were used to calculate the average canopy temperature (Tc), while the air temperature (Ta) was recorded from the AWS at the precise time the image was taken. Finally, Tc and Ta were used to calculate the SDD (°C) [39,40].

### 2.3. Statistical Design and Data Analysis

The experimental design for the trials at Cauquenes and Santa Rosa in seasons 2011 and 2012 was an alpha-lattice with two replicates; for this study, just one replicate (*n* = 384 genotypes) was assessed by thermography in each trial. For seasons 2014 and 2015, the experimental design was a random block with four replicates (16 genotypes; *n* = 64).

Correlations (x versus y) were performed through regression analysis: (1) genotype values: SDD versus GY and Δ^13^C; (2) environmental values: SDD, Tmax, VPDmax, and SWC versus GY and Δ^13^C; and (3) environmental values: VPDmax, Tmax, and SWC versus SDD.

Using the environmental (Tmax, VPDmax, and SWC), phenological (days between stages), physiological (Δ^13^C and SDD) and productive (GY) information (Table 1), a clustering analysis was performed to verify whether the two water regimes evaluated (i.e., FI and WS) were grouped together, within and between seasons and locations, which is important in modeling and validation procedures. This consisted of a series of steps necessary to achieve a correct execution of the analysis methodology. For this study, clustering and hierarchical clustering were used, with the purpose of grouping the different environments studied. A group was defined as the set of elements that have a greater degree of similarity between the objects that belong to the same set [41]. The steps performed in the analysis were the following: obtaining the data, eliminating the columns that do not provide information to the grouping model, normalizing the data, then applying a method of hierarchical clustering using the “ward.D2” method [42] as a grouping form, and plotting the Euclidean distance between elements as a dendrogram. For clustering of groupings, a tree cluster was considered, which uses the Euclidean distance to identify the closeness of the nodes (environmental data points). In addition, this algorithm applies the principal component analysis (PCA) method to show the results with greater clarity [41]. The “ward.D2” was set to find two and three main data groups.

All statistical analysis was performed using R 3.0.0 [43].

## 3. Results

### 3.1. Environmental Conditions, Grain Yield, Carbon Isotope Discrimination, and Stress Degree Days

In general terms, the environmental conditions (Tmax, VPDmax, and SWC) varied according to the seasons, both within and between FI and WS conditions (Table 1, Figures S1 and S2). Under each water supply condition, minimum and maximum values from sowing to harvest were (Table 1): Tmax: 19.1 and 23.5 °C (FI) and 19.1 and 25.4 °C (WS); VPDmax: 1.35 and 1.92 kPa (FI) and 1.35 and 2.39 kPa (WS); and SWC: 198.3–542.7 mm (FI) and 180.4–418.8 mm (WS).

Grain yield under WS conditions was 45% lower than under FI (Table 1). Also, the range of variation among seasons was much greater under WS (1.68–8.13 t ha^−1^) compared to FI (8.03–9.9 t ha^−1^). The Δ^13^C data showed lower values (10.5%) and higher variability under WS conditions compared to FI conditions (Table 1). The average SDD was much higher (5.3 fold) under WS and had greater variability compared to FI conditions (Table 1).

### 3.2. Relationships between Grain Yield and Canopy and Ambient Temperatures in 384 Wheat Genotypes

**Figure 1:**
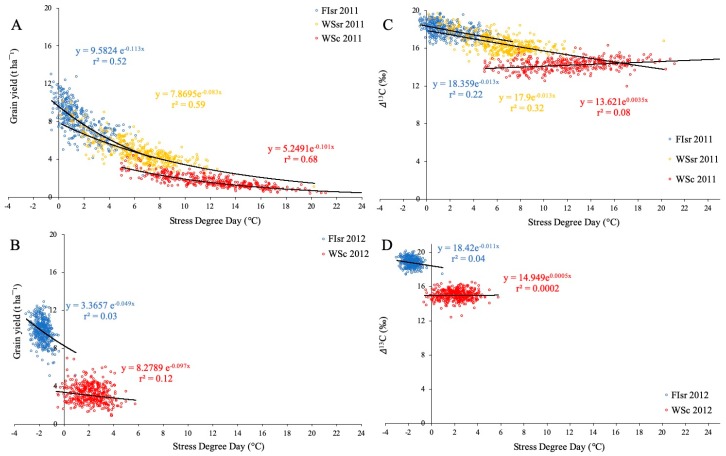
Relationship between stress degree day (SDD = Tc − Ta; where Tc is crop temperature and Ta air temperature, both measured at the soft dough stage (Z85) versus grain yield and carbon isotope discrimination in kernels for 384 spring bread wheat genotypes grown under two water regimes (full irrigation (FI) and water stress (WS)), in two locations (Santa Rosa (sr) and Cauquenes (c)), during the 2011 ((**A**,**C**) respectively) and 2012 seasons ((**B**,**D**), respectively). Regression lines and equations are presented for each water regime and location (determination coefficients are also included).

The relationship between GY and SDD of the 384 genotypes was negative and highly significant in 2011 (r^2^ = 0.52–0.68; *p* < 0.001) (Figure 1A). However, when SSD was compared with Δ^13^C, the determination coefficients (r^2^) were significant only in FIsr and WSsr (0.22 and 0.32, respectively), but not in WSc (Figure 1C). During the second season, r^2^ values were much lower and not significant for both GY (r^2^ = 0.03–0.12; *p* > 0.05) (Figure 1B) and Δ^13^C (r^2^ = 0.0002–0.04; *p* > 0.05) (Figure 1D). In terms of environmental conditions, both seasons showed important differences; Tmax (°C) values were higher in 2011 (WSc = 25.4, FIsr and WSsr = 23.4) than in 2012 (WSc = 20.6 and FIsr = 21.5). Consequently, VPDmax (kPa) in 2011 (WSc = 2.4, FIsr and WSsr = 1.8) was higher than in 2012 (WSc and FIsr = 1.5). In the case of SWC (mm), the values in 2011 (WSc = 381.7, FIsr = 550.7 and WSsr = 507.8) exceeded the values recorded in 2012 (WSc = 256.9 and FIsr = 399.0). Also, SDD (°C) was different between seasons, being higher in 2011 (WSc = 12.3, FIsr = 1.8, and WSsr = 6.4) than in 2012 (WSc = 2.2 and FIsr = −1.7).

### 3.3. Environmental Effects on Grain Yield, Carbon Isotope Discrimination, and Stress Degree Day

**Figure 2:**
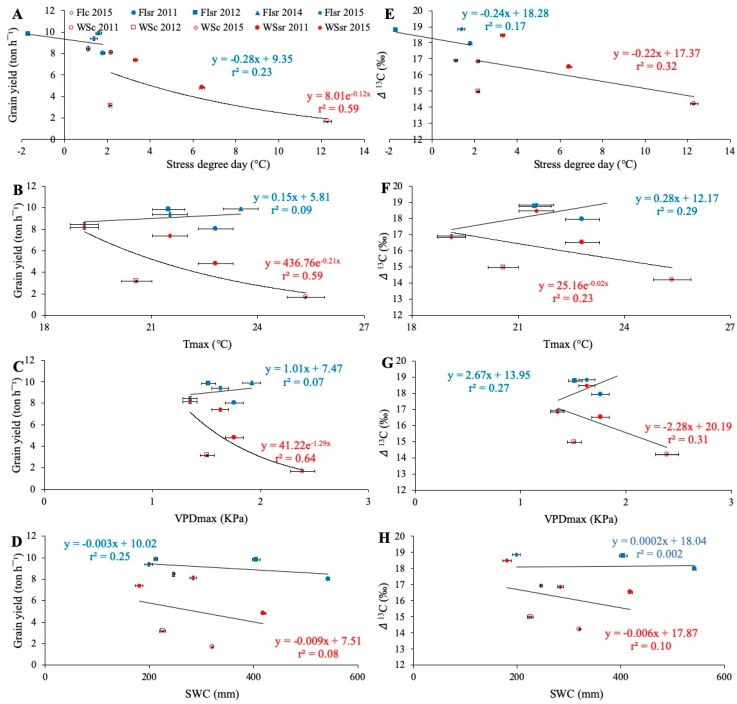
Average grain yield (**A**–**D**) and carbon isotope discrimination in kernels (Δ^13^C; **E**–**H**) of wheat genotypes growing in ten environments, in relation to the stress degree day (SDD = Tc − Ta; Tc is crop temperature and Ta air temperature, both measured at the soft dough stage Z85); **A** and **E**), the seasonal averages of daily maximum temperature (Tmax; **B** and **F**) and maximum vapor pressure deficit (VPDmax; **C** and **G**) and the soil water content between 0 and 50 cm depth (SWC; **D** and **H**). The environments corresponded to the water regime applied (full irrigation (FI) and water stress (WS)), the trial location (Santa Rosa (sr) and Cauquenes (c)), and growing seasons (2011, 2012, 2014, and 2015); the trial code is a combination of these factors. Regression lines and equations are presented for each water regime (determination coefficients are also included).

The average GY of wheat genotypes under FI and WS conditions indicated different responses to environmental variables (Figure 2). Under WS conditions, GY decreased exponentially as SDD, Tmax, and VPDmax increased, which was not the case under FI conditions (Figure 2A–C). Similarly, Δ^13^C also decreased incrementally in SDD, Tmax, and VPDmax (Figure 2E–G). No significant relationships were found between SWC and GY or Δ^13^C (Figure 2D,H).

**Figure 3:**
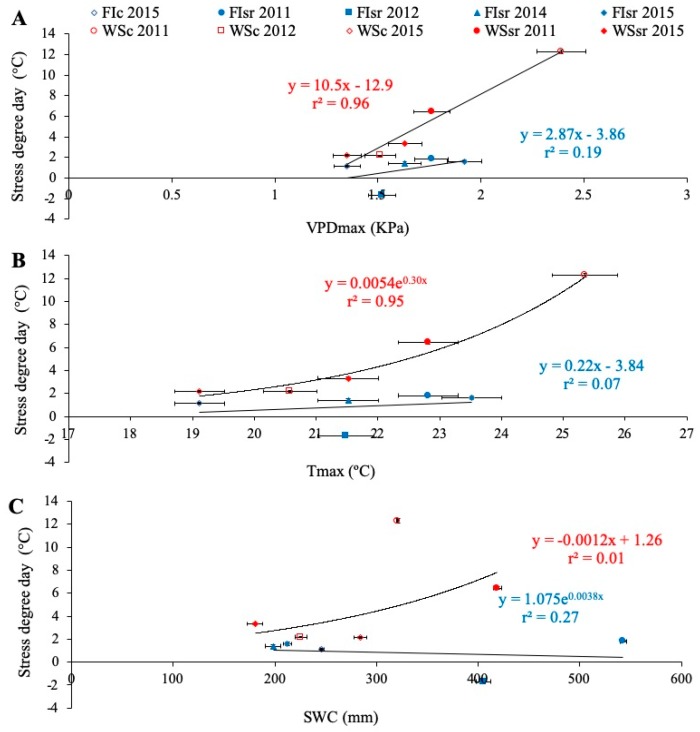
Relationships between the stress degree day (SDD = Tc − Ta; Tc is crop temperature and Ta air temperature, both measured at the soft dough stage Z85), and the seasonal averages of daily maximum vapor pressure deficit (VPDmax) (**A**), maximum temperature (Tmax; **B**) and soil water content between 0 and 50 cm depth (SWC; (**C**)). Mean values were the average of all genotypes growing in the particular environment according to the water regime (full irrigation (FI) and water stress (WS)), the trial location (Santa Rosa (sr) and Cauquenes (c)), and growing seasons (2011, 2012, 2014, and 2015). Regression lines and equations are presented for each water regime (determination coefficients are also included).

In relation to the environmental conditions during the study, when all the environments were combined (Table 2), SDD was only correlated with Tmax (*r* = 0.64; *p* < 0.05) and VPDmax (*r* = 0.80; *p* < 0.01). Under each water regime, close and significant relationships were found between SDD and Tmax and VPDmax, but only in plants growing in WS conditions (Figure 3). The relationship between SDD and SWC was not significant under either WS or FI conditions (Figure 3C).

Pearson correlation analysis (Table 2) showed that mean values of GY in the ten environments were highly correlated with SDD (*r* = −0.81; *p* < 0.01) and Δ^13^C (*r* = 0.92; *p* < 0.01). Also, Δ^13^C was negatively correlated with SDD (*r* = −0.71; *p* < 0.05). In concordance with this, GY and Δ^13^C were primarily affected by Tmax and VPDmax but not by SWC (Figure 2B–F,C–G,D–H, respectively).

**Figure 4:**
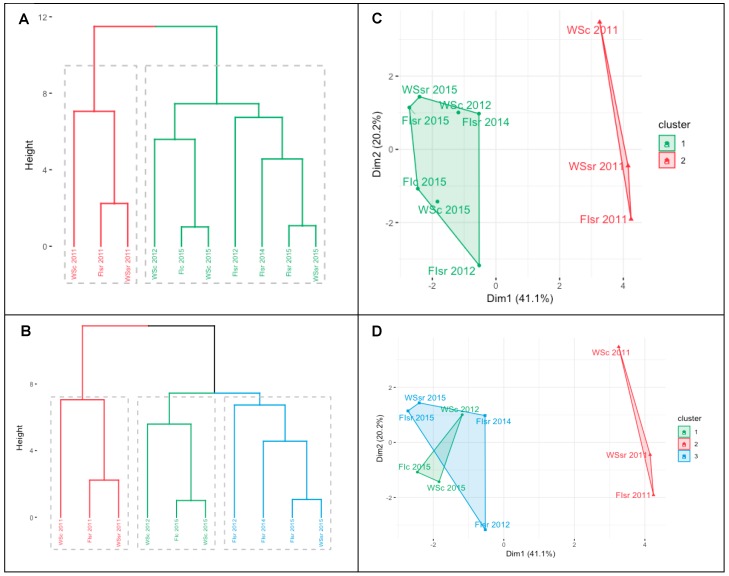
Cluster dendrogram ((**A**) two groups and (**B**) three groups) and plot ((**C**) two groups and (**D**) three groups) for the general characterization of the assessed environments according to the water regime applied (full irrigation (FI) and water stress (WS)), the trial location (Santa Rosa (sr) and Cauquenes (c)), and the evaluated season (2011, 2012, 2014, and 2015); the trial code is a combination of these factors. Data included the phenological (dates), productive (grain yield—GY), physiological (carbon isotope discrimination in kernels (Δ^13^C) and the stress degree day measured at the soft dough stage Z85 (SDD)), and environmental information (seasonal averages of daily maximum temperature and maximum vapor pressure deficit, and the soil water content between 0 and 50 cm depth). In the case of GY, Δ^13^C, and SDD, the mean values analyzed were the average of all genotypes growing in the particular environment.

Finally, when the environmental, phenological, physiological, and productive information (Table 1) was included to generate the two- and three-group cluster dendrograms and plots (Figure 4A,B and Figure 4C,D, respectively), the main difference was found in the division of the first branch of the less stressful environment (green lines in Figure 4A) at the height of the first knot of the most stressful environment (origin of the blue and green lines in Figure 4B). Differences between the cluster plots were according to changes in the cluster dendrograms; the three groups were (Figure 4B): (i) lowest environmental limitations: FIsr 2012, FIsr 2014, FIsr 2015, and WSsr 2015; (ii) intermediate environmental limitations: WSc 2012, Fic 2015, and WSc 2015; and (iii) highest environmental limitations: WSc 2011, FIsr 2011, and WSsr 2011. The cluster plot that explains 61.3% of the variance (Figure 4C) shows a clear distance or separation between the groups with the lowest and the highest environmental constraints (green and red colors in Figure 4, respectively). In the three-group cluster plot, two of the groups overlap. However, even though WSc 2011, FIsr 2011, and WSsr 2011 had the highest SWC values, they also presented, on average, the greatest VPDmax, Tmax and SDD but the lowest GY (Table 1).

## 4. Discussion

### 4.1. Environmental Effects on Grain Yield and Carbon Isotope Discrimination

Tolerance to WS usually implies some improvement or maintenance of metabolic processes that enables the plant to regulate cell water status and maintain leaf turgor under stressful conditions. One of the first mechanisms involved in reducing water loss by transpiration is stomatal control, which partially closes the stomata, thus affecting carbon assimilation and storage [44]. This gas exchange limitation between the atmosphere and the substomatal cavity is primarily driven by the surrounding environmental conditions (e.g., water availability, ambient temperature, relative humidity, wind speed, light intensity). To the extent that the diffusion of CO_2_ through the stomata is more restrictive, the carbon isotope discrimination (Δ^13^C) between ^12^C and ^13^C will also be reduced, increasing the proportion of ^13^C [24]. Therefore, in a particular environment, Δ^13^C at the grain level provides an integrated assessment of the transpiration efficiency during the whole season [25,45]. As in other cereal studies [24,35,46,47,48,49], GY and Δ^13^C in the current work had a strong and positive association (*r* = 0.92) (Table 2). Additionally, the evaluated environmental conditions generated similar responses in GY and Δ^13^C (Figure 2), reaffirming the strong relationship that existed between these characters.

The SWC has also been used as an indicator of water stress in plants and is positively related to GY in wheat [25,50,51,52]. Working in the same species, Reference [53] evaluated the effect of water content in different soil profiles, concluding that a soil that was well irrigated throughout the first 50 cm of the profile obtained a greater yield and harvest index than a soil with dry upper layers. In the present work, even though FI environments always showed higher SWC than under WS in the same location and season (Figure S2A–D), the results did not show a significant correlation between SWC and GY (Table 2). Moreover, while the GY and Δ^13^C were both higher under FI than WS (Figure 2D,H), neither GY nor Δ^13^C were affected by increases in SWC (~200 to 550 mm) under either FI or WS.

Contrastingly, the environmental water demand (VPD; [54]), which is mainly driven by the ambient temperature and relative humidity, proved to influence both GY and Δ^13^C under WS but not in FI (Figure 2C,G). The combination of high temperatures and low relative humidity, which is frequently encountered in the late stages of the growing season in Mediterranean climates (e.g., Santa Rosa and Cauquenes), caused an increase in the VPD. References [55,56] have assessed the effect of environmental variables on wheat physiology and GY, proposing that a high VPD environment should vary between 2.5 and 3.9 kPa. Therefore, the average values of VPDmax found in the present study (1.35 to 2.39 kPa; Figure 2C,G) could be considered moderately low to moderately high, although maximum values reached as high as 6.34 kPa in WSc in 2011 (Figure S1B).

Several studies have proven that growing cereals under non-limiting water conditions but with high VPD values leads to reduced GY and Δ^13^C [21,55,56,57]. The present work, considering all measurements performed, shows a non-significant relationship between GY and VPDmax (Table 2). When FI and WS were analyzed separately, it was only the genotypes growing under WS that showed lower GY and Δ^13^C as VPDmax increased (Figure 2C,G) and the VPD had a higher association with GY than Δ^13^C (Table 2).

Likewise, the Tmax trends were similar to VPDmax (Figure 2B,F). However, despite the Tmax and VPDmax being relatively low (20.6 °C and 1.51 kPa, respectively) in the WSc 2012 trial, the lowest SWC (256.9 mm) was registered, especially after anthesis, and this generated a low GY (3.18 t ha^−1^) (Table 1 and Figure S2B). On the other hand, the WSc 2011 trial had a relatively adequate SWC (320 mm) between anthesis and grain filling (Figure S2B), but due to the late sowing date (Table 1), the plants were exposed to higher Tmax and VPDmax (25.4 °C and 2.39 kPa, respectively) (Table 1), reaching ~40 °C and ~6 kPa for Tmax and VPDmax, respectively (Figure S1A,B), resulting in this trial having the lowest GY (1.68 t ha^−1^).

Under high VPD, guard cell turgor may be decreased by direct evaporative losses from the guard cells and/or decreased water supply to the guard cells if the root or shoot hydraulic conductance is limiting [58], and this leads to a detrimental effect on plant production. Reference [56] tested the effect of VPD and ambient temperature on gas exchange and GY in wheat, finding that environments with high VPD (3.9 kPa) and high temperature (36 °C) increased respiration by up to 22% and decreased photosynthetic water-use efficiency by up to 64% compared to environments with high temperature and lower VPD (1.5 kPa). Indeed, environments with high VPD and temperature caused a reduction in leaf area and net assimilation of CO_2_; however, in the case of plants under the same conditions but without water restriction, there was no decrease in GY. The same authors showed that GY was reduced by 7% in environments with water stress compared to no stress, which is concordant with the findings of the present study, where at similar VPDmax and Tmax (between 1.5 and 2 kPa, and between 21 and 24 °C, respectively), plants grown under WS showed lower GY than plants under FI (Figure 2B,C). In FI conditions, the plants had a GY that was higher than 8 t ha^−1^, while in WS environments, the GY never exceeded that threshold; an exception was WSc 2015 (8.1 t ha^−1^), which was influenced by an abnormally rainy season (“El Niño” phenomenon; Figure S2D).

### 4.2. The Potential of Stress Degree Day to Estimate Grain Yield

Stomatal closure causes a decrease in the transpiration rate, and as a consequence, there is a reduction in the cooling effect, which finally increases leaf/canopy temperature [10]. The reduction in the stomatal conductance could be a consequence of the limitations of the roots to absorb enough water to supply the atmospheric water demand [6]. Numerous studies have confirmed that the temperature of the canopy is associated with crop yield [59,60,61], as well as a series of physiological characteristics, including stomatal conductance [11], the hydric state of the plant [59], and the presence of deep roots.

In general, the present study establishes a negative and highly significant correlation (*r* = −0.81) between SDD and GY (Table 2). Analysis of the responses according to water regime (FI or WS) indicated that there was no significant relationship between GY and SDD in plants growing under FI conditions, but under WS conditions the correlation was moderately high (r^2^ = 0.59) (Figure 2A). The Δ^13^C followed the same pattern, but with lower determination coefficients (FI = 0.17 and WS = 0.34). When Reference [12] studied the relationship between GY and Tc in wheat genotypes grown with similar water regimes (FI and WS), they also found a stronger association under WS (r^2^ = 0.66) than FI (r^2^ = 0.58).

Despite similarities between the studies described above, there are also contradictory results for the relationship between SDD and GY. For example, References [62,63] found no significant relationships, whereas Reference [11] found a high and significant association in irrigated environments. These differences could be explained by the lower VPD registered in the studies of References [62,63] (~2.4 kPa) compared to that of Reference [11] (~5.5 kPa), with the latter case allowing a greater expression of the tolerance of each genotype to the environmental conditions. Although in the present work, there were trials that reached a VPDmax of 6.5 kPa (WSc 2011) (Figure S1), the seasonal averages were ~2.4 kPa.

Therefore, the low SDD values of plants growing under FI is likely due to the ability to meet the water demand of the air (VPD), thus maintaining a high transpiratory rate and allowing the plants to cool down their leaves; under this condition there is more CO_2_ fixation, explaining the higher yields in FI. In this kind of environment where soil water availability is enough to compensate for VPD, the plants do not need to express their water deficit tolerance mechanisms, which in this case means there is a lower SDD versus GY data dispersion, implying lower coefficients of determination.

Similar to the GY and Δ^13^C, SDD was more sensitive to the VPDmax and Tmax than to SWC, with the WS environment having the most effect on plant temperature. Interestingly, an SDD of 2 °C seems to be the threshold between FI and WS environments (Figure 2A,E); SDD averages in FI were lower than 2 °C, while in WS they were greater than 2 °C.

When the relationship between SDD and GY was studied in individual genotypes under contrasting environments (seasons 2011 and 2012), the association (r^2^ values) between these two variables depended on the environment. While in 2011 the relationships in FIsr, WSsr, and WSc were negative and moderately high (r^2^ = 0.52, 0.59, and 0.68, respectively) (Figure 1A), the FIsr and WSc relationships in 2012 were low (r^2^ = 0.03 and 0.12, respectively; WSsr was not sown in 2012) (Figure 1B). As seen before by the use of the average values per environment, the best determination coefficients were observed in more stressful conditions (FI < WSsr < WSc), likely associated with the higher trait-range during the first season; Fisr 2011, WSsr 2011, and WSc 2011 had a higher SDD data dispersion in relation to FIsr 2012 and WSc 2012 (Figure 1 and Figure S1E).

Except for the WSsr 2011 trial, the minimum SDD values of 2011 corresponded, approximately, to the maximums registered during 2012. As already explained, GY is influenced by SDD (Figure 2A,E, respectively), which in turn depends on the VPDmax and Tmax (Figure 3A,B). In this sense, although there was less SWC during the second season, Tmax and VPDmax were lower too, reaffirming that these last two variables would have a more significant impact on the transpiratory and cooling capacity than even the SWC.

Finally, because the main differences in the dendrograms between the two- and three-environment groups were found in the division of the first branch of the less stressful environment (green lines in Figure 4A) at the height of the first knot of the most stressful environment (origin of blue and green lines in Figure 4B), it is logical to think that there were at least three environmental conditions across the trials. In order to establish the possible differences between the superimposed groups (lower and intermediate environmental constraints; green and blue lines in Figure 4D), each trait average was contrasted (percentage of change) with the respective value in the higher environmental limitations group (2011 data; red line in Figure 4D). Using this as a form of normalization, it was possible to establish which traits had the largest and smallest differences between the superimposed groups; GY varied by 52.4% while SDD, Δ^13^C, Tmax, VPDmax, and SWC only varied within the range of 9.77 and 15.1% (data not shown), and these were traits that probably shared similar spatial coordinates in the cluster plot (Figure 4B).

For modeling purposes, it would then be desirable to cease grouping collected data according to text code treatments (e.g., FI and WS according to only the amount of applied water) and start associating them with the environmental conditions existing during the season (e.g., VPDmax). For an adequate estimation of GY and carbon isotope discrimination by thermal imaging, we will then need to use more complex models (e.g., tree-based neural networks) that allow us to identify the “type of environment” in which the collected data should be manipulated to generate and apply the appropriate model. Thus, a deeper environmental characterization would allow development of models with better fit and consistency between years.

## 5. Conclusions and Future Perspectives

The ability to predict GY through the use of thermal images is highly variable and will not only depend on the amount of water stored in the soil profile, but also on other environmental variables such as VPDmax and Tmax. To the extent that better environmental characterization can be achieved, an objective and integral classification of the assessed environment should then be possible. Because the environmental information usually originates from a standard AWS, characterization of the environment at the canopy level or in the first few centimeters above it would also be an important consideration. This would help to generate models with better predictive capacity, thus improving the consistency between seasons.

## Figures and Tables

**Table 1:** Dates of sowing, anthesis, grain filling, and harvest, and mean values of grain yield (GY), carbon isotope discrimination in kernels (Δ^13^C), and stress degree day (SDD = Tc − Ta), determined at the soft dough stage (Z85), for wheat genotypes grown under full irrigation (FI) and water stress (WS) conditions, at Cauquenes (c) and Santa Rosa (sr) in 2011, 2012, 2014, and 2015. Each trial code is a combination of water regime, site, and season. Also, mean values (from sowing to harvest) of daily maximum temperature (Tmax), maximum vapor pressure deficit (VPDmax), and in the case of soil water content (SWC), the average of the daily mean values (from anthesis to mature grain) between 0 and 50 cm depth, are presented.

Trial code	*n*	Dates					Means					
		Sowing	Anthesis	Grain filling	Harvesting		GY (t ha^−1^)	Δ^13^C (‰)	SDD (°C)	Tmax (°C)	VPDmax (kPa)	SWC (mm)
FIsr 2011	384	31 Aug. 2011	24 Nov. 2011	22 Dec. 2011	11 Jan. 2012		8.03	18.0	1.81	23.4	1.76	542.7
FIsr 2012	384	07 Aug. 2012	05 Nov. 2012	19 Nov. 2012	28 Jan. 2013		9.83	18. 8	−1.70	21.5	1.52	404.8
FIsr 2014	64	27 Aug. 2014	24 Nov. 2014	17 Dec. 2014	22 Jan. 2015		9.90	-	1.60	23.5	1.92	212.3
FIsr 2015	64	29 Jul. 2015	20 Nov. 2015	08 Dec. 2015	25 Jan. 2016		9.38	18.8	1.38	21.5	1.63	198.3
FIc 2015	64	18 May 2015	23 Oct. 2015	12 Nov. 2015	23 Dec. 2015		8.46	16.9	1.12	19.1	1.35	246.2
					FI	Average	9.12	18.1	0.84	21.8	1.64	320.9
					FI	SD	0.84	0.90	1.44	1.79	0.22	148.9
					FI	Min.	8.03	16.9	−1.70	19.1	1.35	198.3
					FI	Max.	9.90	18.8	1.81	23.5	1.92	542.7
WSsr 2011	384	31 Aug. 2011	24 Nov. 2011	22 Dec. 2011	11 Jan. 2012		4.81	16.5	6.44	23.4	1.76	418.8
WSc 2011	384	07 Sep. 2011	29 Nov. 2011	13 Dec. 2011	05 Jan. 2012		1.68	14.2	12.29	25.4	2.39	320.9
WSc 2012	384	23 May 2012	11 Sep. 2012	25 Oct. 2012	23 Dec. 2012		3.18	15.0	2.17	20.6	1.51	225.5
WSsr 2015	64	29 Jul. 2015	20 Nov. 2015	08 Dec. 2015	25 Jan. 2016		7.40	18.5	3.31	21.5	1.63	180.4
WSc 2015	64	18 May 2015	23 Oct. 2015	12 Nov. 2015	23 Dec. 2015		8.13	16.9	2.16	19.1	1.35	283.8
					WS	Average	5.04	16.2	5.27	22.0	1.74	285.9
					WS	SD	2.45	1.49	4.29	2.18	0.36	91.8
					WS	Min.	1.68	14.2	2.16	19.1	1.35	180.4
					WS	Max.	8.13	18.5	12.3	25.4	2.39	418.8

**Table 2:** Pearson’s correlation matrix for stress degree day (SDD = Tc − Ta; Tc is crop temperature and Ta air temperature, both measured at the soft dough stage Z85), grain yield (GY), carbon isotope discrimination in kernels (Δ^13^C), and seasonal averages of daily maximum temperature (Tmax), maximum vapor pressure deficit (VPDmax), and soil water content between 0 and 50 cm depth (SWC; between anthesis and mature grain). Data from the water regime applied (full irrigation and water stress), the trial location (Santa Rosa and Cauquenes), and the evaluated season (2011, 2012, 2014, and 2015).

**GY**	−0.81 **	-	-	-	-
**Δ^13^C**	−0.71 *	0.92 **	-	-	-
**Tmax**	0.64 *	−0.38	−0.16	-	-
**VPDmax**	0.80 **	−0.52	−0.35	0.95 **	-
**SWC**	0.06	−0.06	−0.05	0.29	0.16
	**SDD**	**GY**	**Δ^13^C**	**Tmax**	**VPDmax**

* Statistically significant (*p* < 0.05); ** Highly statistically significant (*p* < 0.01).

## Supplementary Materials

The following are available online at https://www.mdpi.com/1424-8220/19/12/2676/s1, Figure S1: (**A**) Canopy temperature—air temperature (SDD; (A) at the early dough stage of grain filling (Zadoks Z83); (**B**) grain yield; (**C**) daily maximum temperature (from sowing to harvest; carbon isotope discrimination (Δ^13^C) in kernels; and (**D**) daily maximum VPD from sowing to harvest, for genotypes of wheat grown under full irrigation (FI) and water stress (WS) at Santa Rosa (sr) and Cauquenes (c) during four growing seasons (2011, 2012, 2014, and 2015). Box and whiskers show minimum, 25th percentile, median, mean, 75th percentile, and maximum values. Open symbols represent outlier data; Figure S2: Soil water content between 0 and 50 cm depth and precipitation according to the water regime applied (full irrigation (FI) and water stress (WS)), the trial location (Santa Rosa (sr) and Cauquenes (c)), and the evaluated seasons: 2011 (**A**), 2012 (**B**), 2014 (**C**), and 2015 (**D**); the trial code is a combination of these factors. Bars represent the precipitation and the arrows the phenological stages at Santa Rosa (orange) and Cauquenes (black). Solid arrows indicate anthesis and dashed arrows the early dough stage (Z85 from the Zadoks scale).

## References

[1] Tanner C.B. (1963). Plant Temperatures 1. Agron. J..

[2] Zia S., Spohrer K., Merkt N., Wenyong D., He X., Müller J. (2009). Non-invasive water status detection in grapevine (*Vitis vinifera* L.) by thermography. Int. J. Agric. Biol. Eng..

[3] Jones H.G., Stoll M., Santos T., Sousa C.D., Chaves M.M., Grant O.M. (2002). Use of infrared thermography for monitoring stomatal closure in the field: Application to grapevine. J. Exp. Bot..

[4] Leinonen I., Jones H.G. (2004). Combining thermal and visible imagery for estimating canopy temperature and identifying plant stress. J. Exp. Bot..

[5] Möller M., Alchanatis V., Cohen Y., Meron M., Tsipris J., Naor A., Cohen S. (2007). Use of thermal and visible imagery for estimating crop water status of irrigated grapevine. J. Exp. Bot..

[6] Wang W.S., Pan Y.J., Zhao X.Q., Dwivedi D., Zhu L.H., Ali J., Li Z.K. (2010). Drought-induced site-specific DNA methylation and its association with drought tolerance in rice (*Oryza sativa* L.). J. Exp. Bot..

[7] Zia S., Spohrer K., Wenyong D., Spreer W., Romano G., Xiongkui H., Joachim M. (2011). Monitoring physiological responses to water stress in two maize varieties by infrared thermography. Int. J. Agric. Biol. Eng..

[8] Jackson R.D., Idso S.B., Reginato R.J., Pinter P.J. (1981). Canopy temperature as a crop water stress indicator. Water Resour. Res..

[9] Luquet D., Bégué A., Vidal A., Clouvel P., Dauzat J., Olioso A., Tao Y. (2003). Using multidirectional thermography to characterize water status of cotton. Remote Sens. Environ..

[10] Jones H.G. (2004). Application of thermal imaging and infrared sensing in plant physiology and ecophysiology. Adv. Bot. Res..

[11] Amani I., Fischer R.A., Reynolds M.P. (1996). Canopy temperature depression association with yield of irrigated spring wheat cultivars in a hot climate. J. Agron. Crop Sci..

[12] Cossani C.M., Pietragalla J., Reynolds M., Reynolds M.P., Pask A.J.D., Mullan D.M., Chávez-Dulanto P.N. (2013). Temperatura del dosel vegetal y características de la relación planta-agua. Fitomejoramiento fisiológico I: Enfoques interdisciplinarios para mejorar la adaptación del cultivo.

[13] Fischer R.A., Rees D., Sayre K.D., Lu Z.M., Condon A.G., Saavedra A.L. (1998). Wheat yield progress associated with higher stomatal conductance and photosynthetic rate, and cooler canopies. Crop Sci..

[14] Leinonen I., Grant O.M., Tagliavia C.P.P., Chaves M.M., Jones H.G. (2006). Estimating stomatal conductance with thermal imagery. Plant Cell Environ..

[15] Grant O.M., Tronina Ł., Jones H.G., Chaves M. (2007). Exploring thermal imaging variables for the detection of stress responses in grapevine under different irrigation regimes. J. Exp. Bot..

[16] Prashar A., Jones H.G. (2014). Infra-red thermography as a high-throughput tool for field phenotyping. Agronomy.

[17] Prashar A., Jones H.G., Duque P. (2016). Assessing drought responses using thermal infrared imaging. Environmental Responses in Plants. Methods and Protocols, Methods in Molecular Biology.

[18] Blum A., Shpiler L., Golan G., Mayer J. (1989). Yield stability and canopy temperature of wheat genotypes under drought-stress. Field Crops Res..

[19] Condon A.G., Richards R.A., Farquhar G.D. (1987). Carbon isotope discrimination is positively correlated with grain yield and dry matter production in field-grown wheat. Crop Sci..

[20] Farquhar G.D., Hubick K.T., Condon A.G., Richards R.A., Rundel P.W., Ehleringer J.R., Nagy K.A. (1989). Carbon isotope fractionation and plant water-use efficiency. Stable Isotopes in Ecological Research.

[21] Condon A.G., Richards R.A., Farquhar G.D. (1992). The effect of variation in soil water availability, vapour pressure deficit and nitrogen nutrition on carbon isotope discrimination in wheat. Aust. J. Agric. Res..

[22] Araus J.L., Amaro T., Casadesus J., Asbati A., Nachit M.M. (1998). Relationships between ash content, carbon isotope discrimination and yield in durum wheat. Funct. Plant Biol..

[23] Araus J.L., Villegas D., Aparicio N., Del Moral L.F., El Hani S., Rharrabti Y., Royo C. (2003). Environmental factors determining carbon isotope discrimination and yield in durum wheat under Mediterranean conditions. Crop Sci..

[24] Lobos G.A., Matus I., Rodriguez A., Romero-Bravo S., Araus J.L., del Pozo A. (2014). Wheat genotypic variability in grain yield and carbon isotope discrimination under mediterranean conditions assessed by spectral reflectance. J. Integr. Plant Biol..

[25] Cabrera-Bosquet L., Albrizio R., Nogués S., Araus J.L. (2010). Dual Δ^13^C/δ^18^O response to water and nitrogen availability and its relationship with yield in field-grown durum wheat. Plant Cell Environ..

[26] Garriga M., Romero-Bravo S., Estrada F., Escobar A., Matus I., del Pozo A., Astudillo C., Lobos G. (2017). Assessing wheat traits by spectral reflectance: Do we really need to focus on predicted trait-values or directly identify the elite genotypes group?. Front. Plant Sci..

[27] Moretti C.L., Mattos L.M., Calbo A.G., Sargent S.A. (2010). Climate changes and potential impacts on postharvest quality of fruit and vegetable crops: A review. Food Res. Int..

[28] Lobos G.A., Hancock J.F. (2015). Breeding blueberries for a changing global environment: A review. Front. Plant Sci..

[29] Camargo A.V., Lobos G.A. (2016). Latin America: A development pole for phenomics. Front. Plant Sci..

[30] Lobos G.A., Camargo A.V., del Pozo A., Araus J.L., Ortiz R., Doonan J.H. (2017). Plant Phenotype and phenomics for plant breeding. Front. Plant Sci..

[31] Sadras V.O., Reynolds M.P., De la Vega A.J., Petrie P.R., Robinson R. (2009). Phenotypic plasticity of yield and phenology in wheat, sunflower and grapevine. Field Crops Res..

[32] Mora F., Castillo D., Lado B., Matus I., Poland P., Belzile F., von Zitzewitz J., del Pozo A. (2015). Genome-wide association mapping of agronomic traits and carbon discrimination in a worldwide germplasm collection of spring wheat using SNP markers. Mol. Breed..

[33] del Pozo A., Yáñez A., Matus I.A., Tapia G., Castillo D., Sanchez-Jardón L., Araus J.L. (2016). Physiological traits associated with wheat yield potential and performance under water-stress in a mediterranean environment. Front. Plant Sci..

[34] Zhang D., Du Q., Zhang Z., Jiao X., Song X., Li J. (2017). Vapour pressure deficit control in relation to water transport and water productivity in greenhouse tomato production during summer. Sci. Rep..

[35] Hernández J., Lobos G.A., Matus I., del Pozo A., Silva P., Galleguillos M. (2015). Using ridge regression models to estimate grain yield from field spectral data in bread wheat (*Triticum aestivum* L.) grown under three water regimes. Remote Sens..

[36] Zadoks J.C., Chang T.T., Konzak C.F. (1974). A decimal code for the growth stages of cereals. Weed Res..

[37] Allen R.G., Pereira L.S., Raes D., Smith M. (1998). Crop Evapotranspiration: Guidelines for Computing Crop Water Requirements. FAO Irrigation and Drainage Paper N° 56.

[38] Fuentes S., De Bei R., Pech J., Tyerman S. (2012). Computational water stress indices obtained from thermal image analysis of grapevine canopies. Irrig. Sci..

[39] Idso S.B., Jackson R.D., Reginato R.J. (1977). Remote sensing of crop yields. Science.

[40] Jackson R.D., Reginato R.J., Idso S.B. (1977). Wheat canopy temperature: A practical tool for evaluating water requirements. Water Resour. Res..

[41] Nerurkar P., Shirke A., Chandane M., Bhirud S. (2018). Empirical analysis of data clustering algorithms. Procedia Comput. Sci..

[42] Murtagh F., Legendre P. (2014). Ward’s hierarchical agglomerative clustering method: Which algorithms implement ward’s criterion?. J. Classif..

[43] R Development Core Team (2011). R: A Language and Environment for Statistical Computing.

[44] Ryan A.C., Dodd I.C., Rothwell S.A., Jones R., Tardieu F., Draye X., Davies W.J. (2016). Gravimetric phenotyping of whole plant transpiration responses to atmospheric vapour pressure deficit identifies genotypic variation in water use efficiency. Plant Sci..

[45] Farquhar G.D., O’Leary M.H., Berry J.A. (1982). On the relationship between carbon isotope discrimination and the intercellular carbon dioxide concentration in leaves. Funct. Plant Biol..

[46] Acevedo E.H., Baginsky C.G., Solar B.R., Ceccarelli S. (1997). Discriminación isotópica de C13 y su relación con el rendimiento y la eficiencia de transpiracion de genotipos locales y mejorados de cebada bajo diferentes condiciones hídricas. Inv. Agric..

[47] Rebetzke G.J., Condon A.G., Farquhar G.D., Appels R., Richards R.A. (2008). Quantitative trait loci for carbon isotope discrimination are repeatable across environments and wheat mapping populations. Theor. Appl. Genet..

[48] del Pozo A., Castillo D., Inostroza L., Matus I., Méndez A.M., Morcuende R. (2012). Physiological and yield responses of recombinant chromosome substitution lines of barley to terminal drought in a mediterranean type environment. Ann. Appl. Biol..

[49] Araus J.L., Cabrera-Bosquet L., Serret M.D., Bort J., Nieto-Taladriz M.T. (2013). Comparative performance of δ^13^C, δ^18^O and δ^15^N for phenotyping durum wheat adaptation to a dryland environment. Funct. Plant Biol..

[50] Passioura J.B. (1977). Grain yield, harvest index, and water use of wheat. J. Aust. Inst. Agric. Sci..

[51] French R.J., Schultz J.E. (1984). Water use efficiency of wheat in a Mediterranean-type environment. I. The relation between yield, water use and climate. Aust. J. Agric. Res..

[52] Sun H.Y., Liu C.M., Zhang X.Y., Shen Y.J., Zhang Y.Q. (2006). Effects of irrigation on water balance, yield and WUE of winter wheat in the north China plain. Agric. Water Manag..

[53] Li F.M., Liu X.L., Li S.Q. (2001). Effects of early soil water distribution on the dry matter partition between roots and shoots of winter wheat. Agric. Water Manag..

[54] Anderson D.B. (1936). Relative humidity or vapor pressure deficit. Ecology.

[55] Dreccer M.F., Fainges J., Whish J., Ogbonnaya F.C., Sadras V.O. (2018). Comparison of sensitive stages of wheat, barley, canola, chickpea and field pea to temperature and water stress across Australia. Agric. For. Meteorol..

[56] Rashid M.A., Andersen M.N., Wollenweber B., Zhang X., Olesen J.E. (2018). Acclimation to higher VPD and temperature minimized negative effects on assimilation and grain yield of wheat. Agric. For. Meteorol..

[57] Sharifi M.R., Rundel P.W. (1993). The effect of vapour pressure deficit on carbon isotope discrimination in the desert shrub *Larrea tridentata* (creosote bush). J. Exp. Bot..

[58] Franks P.J. (2004). Stomatal control and hydraulic conductance, with special reference to tall trees. Tree Physiol..

[59] Blum A., Mayer J., Gozlan G. (1982). Infrared thermal sensing of plant canopies as a screening technique for dehydration avoidance in wheat. Field Crops Res..

[60] Reynolds M.P., Balota M., Delgado M.I.B., Amani I., Fischer R.A. (1994). Physiological and morphological traits associated with spring wheat yield under hot, irrigated conditions. Funct. Plant Biol..

[61] Olivares-Villegas J.J., Reynolds M.P., McDonald G.K. (2007). Drought-adaptive attributes in the Seri/Babax hexaploid wheat population. Funct. Plant Biol..

[62] Idso S.B., Reginato R.J., Clawson K.L., Anderson M.G. (1984). On the stability of non-water-stressed baselines. Agric. For. Meteorol..

[63] Hatfield J.L. (1990). Measuring plant stress with an infrared thermometer. HortScience.

