# Supplementary material for: Thermal Imaging Reliability for Estimating Grain Yield and Carbon Isotope Discrimination in Wheat Genotypes: Importance of the Environmental Conditions
Source: Sensors (Basel). 2019 Jun 13;19(12):2676. doi: 10.3390/s19122676 (PMC6630921; doi:10.3390/s19122676)
Supplement: Supplementary file 1 [file sensors-19-02676-s001.pdf]

Supplementary information

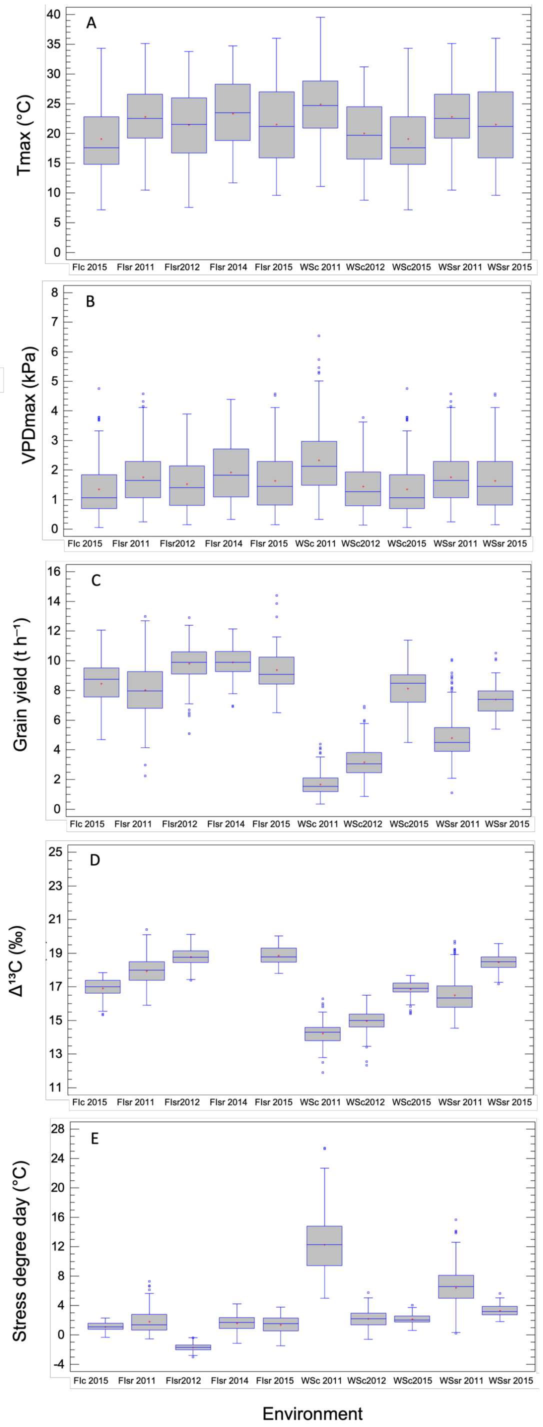

Supplementary Figure 1. A) Canopy temperature – air temperature (SDD; A) at grain filling early dough stage (Zadoks Z83); B) grain yield; C) daily maximum temperature (from sowing to harvest; carbon isotope discrimination ($\Delta^{13}C$) in kernels; and D) daily maximum VPD from sowing to harvest, for genotypes of wheat grown under full irrigation (FI) and water stress (WS) at Santa Rosa (sr) and Cauquenes (c) in four growing seasons (2011, 2012, 2014 and 2015). Box and whiskers show minimum, 25th percentile, median, mean, 75th percentile and maximum values. Open symbols represent outlier data.

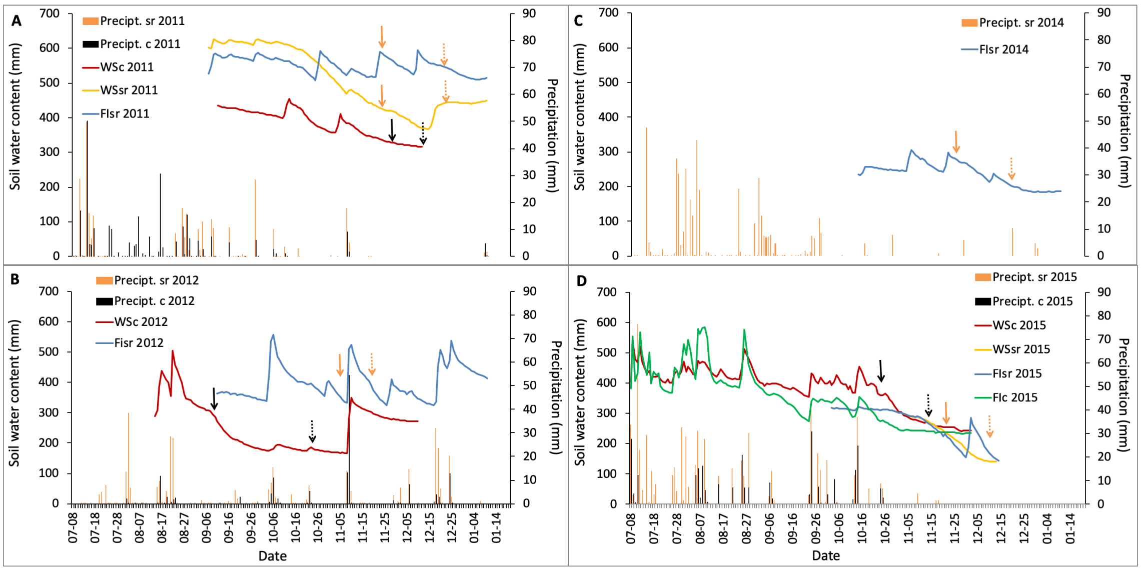

Supplementary Figure 2. Soil water content between 0 and 50 cm depth and precipitation according to the water regime applied (full irrigation – FI and water stress – WS), the trial location (Santa Rosa – sr and Cauquenes – c), and the evaluated seasons: 2011 (A), 2012 (B), 2014 (C), and 2015 (D); the trial code is a combination of these factors. Bars represent the precipitation and the arrows the phenological stages at Santa Rosa (orange) and Cauquenes (black). Solid arrows indicate anthesis and dashed arrows the grain filling early dough stage (Z83 from the Zadoks scale).
